# Isolating the sources of pipeline‐variability in group‐level task‐fMRI results

**DOI:** 10.1002/hbm.25713

**Published:** 2021-11-13

**Authors:** Alexander Bowring, Thomas E. Nichols, Camille Maumet

**Affiliations:** ^1^ Li Ka Shing Centre for Health Information and Discovery, Nuffield Department of Population Health Big Data Institute, University of Oxford Oxford UK; ^2^ Wellcome Centre for Integrative Neuroimaging, FMRIB, Nuffield Department of Clinical Neurosciences University of Oxford Oxford UK; ^3^ Department of Statistics University of Warwick Coventry UK; ^4^ Inria, Univ Rennes, CNRS, Inserm, IRISA UMR 6074, Empenn ERL U 1228 Rennes France

**Keywords:** AFNI, analytic flexibility, analytic variability, fMRI, FSL, reproducibility, software comparison, SPM, task‐fMRI

## Abstract

Task‐fMRI researchers have great flexibility as to how they analyze their data, with multiple methodological options to choose from at each stage of the analysis workflow. While the development of tools and techniques has broadened our horizons for comprehending the complexities of the human brain, a growing body of research has highlighted the pitfalls of such methodological plurality. In a recent study, we found that the choice of software package used to run the analysis pipeline can have a considerable impact on the final group‐level results of a task‐fMRI investigation (Bowring et al., 2019, *BMN*). Here we revisit our work, seeking to identify the stages of the pipeline where the greatest variation between analysis software is induced. We carry out further analyses on the three datasets evaluated in *BMN*, employing a common processing strategy across parts of the analysis workflow and then utilizing procedures from three software packages (AFNI, FSL, and SPM) across the remaining steps of the pipeline. We use quantitative methods to compare the statistical maps and isolate the main stages of the workflow where the three packages diverge. Across all datasets, we find that variation between the packages' results is largely attributable to a handful of individual analysis stages, and that these sources of variability were heterogeneous across the datasets (e.g., choice of first‐level signal model had the most impact for the balloon analog risk task dataset, while first‐level noise model and group‐level model were more influential for the false belief and antisaccade task datasets, respectively). We also observe areas of the analysis workflow where changing the software package causes minimal differences in the final results, finding that the group‐level results were largely unaffected by which software package was used to model the low‐frequency fMRI drifts.

## Graphical abstract

In this article, we seek to identify the stages of the pipeline where the greatest variation between analysis software is induced. We carry out further analyses on the three datasets evaluated in Bowring, Maumet, and Nichols, 2019, employing a common processing strategy across parts of the analysis workflow and then utilizing procedures from AFNI, FSL, and SPM across the remaining steps of the pipeline. Across all datasets, we find that variation between the packages' results is largely attributable to a handful of individual analysis stages, and that these sources of variability were heterogeneous across the datasets.

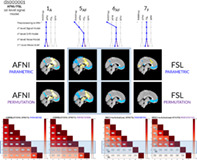

## 1 INTRODUCTION

The 2010's may be best remembered by scientists as the start of the “replication crisis” (Maxwell, Lau, & Howard, 2015), an ongoing issue that has gained prominence as a number of classic and contemporary psychology studies have been brought into question. At the heart of the controversy is a growing body of work where attempts to replicate several effects in psychological science have failed, prompting further scrutiny of the robustness of the original findings. In a landmark investigation, the Open Science Collaboration (2015) repeated 100 experiments that had been published in three high‐ranking psychology journals, reporting that only 36% of their replications determined a positive result compared to 97% of the original studies. At around the same time the first in a series of Many Labs studies was published, where numerous analysis teams have tried to replicate results from the psychology literature across a diverse range of samples. Of the 51 studies re‐evaluated in the first three Many Labs projects, roughly 60% yielded significant effects (Ebersole et al., 2016; Klein et al., 2014, 2018).

The field of functional magnetic resonance imaging (fMRI) for human brain mapping has not come away unharmed from the replication crisis. On the contrary, the large degree of flexibility in neuroimaging analysis workflows has been pinpointed as an aspect of the field that can hinder reproducibility (Ioannidis, 2005). The crux of the problem is that two different analysis pipelines applied to the same dataset are unlikely to give the same result. Therefore, as an increasing number of analytical tools and techniques have become available to researchers, this has also increased the potential to yield distorted findings with inflated levels of false activations. When combined with selective reporting practices—where only methods that return a favorable outcome are likely to end up being published—the consequences of this can be severe, leading to fMRI effect sizes that are misrepresented and often overstated in the neuroimaging literature (Simmons, Nelson, & Simonsohn, 2011; Szucs & Ioannidis, 2017).

In one of the most comprehensive studies in this area, a single publicly available fMRI dataset was analyzed using over 6,000 unique simulated workflows, constructed by enumerating all possible pipeline combinations from an array of commonly implemented analysis procedures (Carp, 2012). Across the tens of thousands of thresholded results maps generated by these workflows, a substantial degree of variability was observed in both the sizes and locations of significant activation. In a more recent study, 70 independent research teams were tasked with testing 9 hypotheses on the same fMRI dataset, with no constraints placed on how each team approached their analysis (Botvinik‐Nezer et al., 2020). Consequently, no two teams chose the same analysis workflow, and once again, the plurality of methodological approaches manifested as variability in the final scientific outcomes, this time with considerable disagreement between the 70 teams' hypothesis test results. Overall, these investigations have forewarned practitioners not to fall victim to a version of insanity where we apply *different* workflows over and over again and expect *the same* results.

In Bowring, Maumet, and Nichols (2019) (*BMN*), we discovered that it is not just the procedures comprising the analysis pipeline that can induce variation across fMRI results, but also the choice of *software package* through which the analysis is conducted. We reanalyzed three datasets connected to three published task‐fMRI studies within the three most widely‐used neuroimaging software packages—AFNI (Cox, 1996; Cox & Hyde, 1997), FSL (Jenkinson et al., 2012), and SPM (Penny, Friston, Ashburner, Kiebel, & Nichols, 2011)—reproducing the original publication's analysis workflows in each package as closely as possible so that the difference in software was the only changing variable. We then applied a range of similarity metrics to quantify the differences between each software's final group‐level results. While qualitatively certain patterns of signal were observed across all three packages' statistical results maps, our quantitative comparisons displayed marked differences in the size, magnitude, and topology of activated brain regions, and we ultimately concluded that weak effects may not generalize across software.

Now we revisit that work, seeking to understand *where* in the analysis pipeline the greatest variation between analysis software is induced. We substantially extend the analyses carried out for BMN, running the same three datasets through a series of “hybrid” pipelines that employ a common processing strategy across parts of the workflow (e.g., by implementing a common fMRIPrep preprocessing strategy) and then interchange pipeline elements between software for the remaining stages of the analysis. By comparing all sets of our analysis results, we isolate the key stages of the workflow where the three packages diverge. Ultimately, we find that the variation between the packages' results is largely attributable to sizable processing differences at a handful of key analysis stages, and that these sources of variability can be heterogeneous across datasets. Finally, for each study we apply an image‐based meta‐analysis procedure recently used in Botvinik‐Nezer et al. (2020) to all of our analysis results, aggregating the information acquired from running one dataset through multiple pipelines to obtain a consensus map of activated brain regions.

The remainder of the manuscript is organized as follows: First, we provide a brief summary of the three original published studies from which we sourced our selected datasets. We then describe the pipelines implemented for our reanalyses of the data, and detail the quantitative and qualitative metrics and image‐based meta‐analysis procedure applied to our analysis results. Finally, we evaluate our findings to assess the magnitude of variation between fMRI analysis software at each stage of the analysis workflow, and discuss the repercussions of these results on the functional neuroimaging literature.

## 2 METHODS

We first provide an overview of the original study paradigms for the three published task‐fMRI works from which we sourced the three datasets, before we go on to detail the reanalysis methods carried out in this work. Most notably, while the original studies' analyses were carried out on 16, 29, and 30 participants task‐fMRI data respectively, for the latter two studies only 21 and 17 participants' data were available for reanalyses. Alongside this, due to preprocessing failing for one individual in the ds000001 dataset, we ultimately reanalyzed 15 subjects rather than the complete sample of 16 whose data were shared (see the start of Section 3 for more details).

### 2.1 Study description and data source

We selected three task‐fMRI studies from the publicly accessible OpenfMRI (now upgraded to OpenNeuro, RRID:SCR_005031) data repository (Gorgolewski, Esteban, Schaefer, Wandell, & Poldrack, 2017), OpenfMRI dataset accession numbers: ds000001 (Revision: 2.0.4; Schonberg et al., 2012), ds000109 (Revision 2.0.2; Moran, Jolly, & Mitchell, 2012), and ds000120 (Revision 1.0.0; Padmanabhan, Geier, Ordaz, Teslovich, & Luna, 2011). Each of the datasets had been organized in compliance with the Brain Imaging Data Structure (BIDS, RRID:SCR_016124; Gorgolewski et al., 2016). These datasets were chosen following an extensive selection procedure (carried out between May 2016 and November 2016), whereby we vetted the associated publication for each dataset stored in the repository. We sought to find studies with simple analysis pipelines and clearly reported regions of brain activation that would be easily comparable to our own results. Exclusion criteria included the use of custom software, activations defined using small volume correction, and application of more intricate methods such as region of interest and robust regression analysis, which we believed could be impractical to implement across all analysis software. A full description of the paradigm for each of our chosen studies is included in the respective publication, here we give a brief overview.

For the ds000001 study, 16 healthy adult subjects participated in a balloon analog risk task over three scanning sessions. On each trial, subjects were presented with a simulated balloon, and offered a monetary reward to “pump” the balloon. With each successive pump the money would accumulate, and at each stage of the trial subjects had a choice of whether they wished to pump again or cash‐out. After a certain number of pumps, which varied between trials, the balloon exploded. If subjects had cashed‐out before this point they were rewarded with all the money they had earned during the trial, however if the balloon exploded all money accumulated was lost. Three different colored “reward” balloons were used between trials, each having a different explosion probability, as well as a gray “control” balloon, which had no monetary value and would disappear from the screen after a predetermined number of pumps. Here we reproduce the pipeline used to obtain the main study result contrasting the parametrically modulated activations of pumps of the reward balloons versus pumps of the control balloon, corresponding to Figure 3 and Table 2 in the original article. Group‐level inference was performed using an uncorrected cluster‐forming threshold *p <*.01, FWE‐corrected clusterwise threshold *p <*.05.

The ds000109 study investigated the ability of people from different age‐groups to understand the mental state of others. A total of 48 subjects participated, although imaging data was obtained from only 43 participants for the false belief task: 29 younger adults and 14 older adults. In this task participants listened to either a “false belief” or “false photo” story. A false belief story would entail an object being moved from one place to another, with certain characters witnessing the change in location while others were unaware. False photo stories were similar except that they involved some physical representation of the missing object, such as a photo of an object in a location from which it had been subsequently removed. The task had a block design where stories were represented for 10s, after which participants had to answer a question about one of the character's perceptions of the location of the object. We reproduce the pipeline used to obtain the contrast map of false belief versus false photo activations for the younger adults, corresponding to Figure 5a and Table 3 from the original publication. Group‐level inference was performed using an uncorrected cluster‐forming threshold *p <*.005, FWE‐corrected clusterwise threshold *p* <.05.

Finally, the ds000120 study explored reward processing across different age groups. fMRI results were reported on 30 subjects, with 10 participants belonging to each of the three age groups (children, adolescents, and adults). Participants took part in an antisaccade task where a visual stimulus was presented in each trial and subjects were instructed to quickly fixate their gaze on the side of the screen opposite to the stimulus. Prior to a trial, subjects were given a visual cue to signal whether or not they had the potential to win a monetary reward based on their upcoming performance (a “reward” or “neutral” trial). We reproduce the pipeline used to obtain the main effect of time activation map, an *F*‐statistic for any nonzero coefficients in the sine HRF basis, corresponding to Figure 3 and Table 1 in the original publication. Group‐level inference was performed using an uncorrected cluster‐forming threshold *p <*.001, FWE‐corrected clusterwise threshold *p <*.05.

**TABLE 1:** fMRIPrep processing pipeline

Workflow	Processing step	Description	Tools used
Structural preprocessing	Nonuniform intensity correction	The anatomical T1w image was corrected for intensity nonuniformity with N4BiasFieldCorrection, distributed within ANTs 2.2.0, to be used as the anatomical reference image for the rest of the pipeline.	ANTS
	Brain extraction	The anatomical reference image was skull‐stripped with a Nipype implementation of the antsBrainExtraction.sh workflow from ANTs.	ANTS
	Segmentation	Brain tissue segmentation of the CSF, WM, and GM was performed on the brain‐extracted T1w using FSL's fast.	FSL
	Brain surface reconstruction	Brain surfaces were reconstructed using FreeSurfer's recon‐all, after which the brain mask was refined using a custom variation of the ANTs‐derived and FreeSurfer‐derived segmentations of the cortical GM.	FreeSurfer, ANTS
	T1w‐to‐MNI152 registration	Spatial normalization to MNI152 space was performed through nonlinear registration with ANTs' antsRegistration, using brain‐extracted versions of the T1w reference and template images.	ANTS
Functional preprocessing	Reference image	For each BOLD run, a custom methodology of fMRIPrep was applied to average across the BOLD time‐series in order to generate a reference volume.	Custom
	Brain extraction	The BOLD reference image was skull‐stripped using NiWorkflows' init_enhance_and_skullstrip_BOLD_wf(), to be used for head‐motion estimation and registration of the BOLD time‐series images to the subject's T1w image.	NiWorkflows
	BOLD‐to‐T1w registration	The BOLD reference was co‐registered to the T1w reference using FreeSurfer's bbregister, implementing a boundary‐based registration with six degrees of freedom.	FreeSurfer
	Head‐motion correction	Head‐motion parameters with respect to the BOLD reference (one rigid‐body transformation, three rotations and three translations) were estimated with FSL's mcflirt, after which the rigid‐body transformation was applied to re‐sample the BOLD time‐series onto their original, native space.	FSL
	BOLD‐to‐MNI152 registration	Transformations already obtained (head‐motion rigid‐body transformation, BOLD to T1w registration, T1w to MNI152 transformation) were concatenated to map the BOLD image to the MNI152 standard space.	Smooth
	Confound estimation	A range of potential confounds were estimated, including the mean global signal, mean tissue class signal, tCompCor, aCompCor, Framewise Displacement, and DVARS.	CompCo

For all three studies, a subset of the workflows applied the same fMRIPrep preprocessing pipeline. Here, we itemize the main steps of the fMRIPrep preprocessing pipeline, making note of the various tools used at each stage of the workflow.

### 2.2 Previous analyses and preprocessing methods

In BMN we reanalyzed the ds000001 and ds000109 studies described in the previous section using each of the three software packages: AFNI (version AFNI_18.1.09; Cox, 1996, Cox & Hyde, 1997), RRID:SCR_005927; FSL (version 5.0.10, Jenkinson et al., 2012), RRID:SCR_002823; and SPM (version SPM12, v6906; Penny et al., 2011), RRID:SCR_007037. For ds000120, the repeated‐measures design carried out for the original group‐level analysis was not feasible to implement in FSL; while the manual for FSL's fMRI Expert Analysis Tool (FEAT) describes “Repeated Measures” examples, these are based on a restrictive assumption of compound symmetry that would entail assuming all 28 correlations among the basis regression coefficients are equal. Because of this, for ds000120 we reanalyzed the data in AFNI and SPM only. In parallel to our reproductions of the original analysis workflows, for ds000001 and ds000109 we computed an additional set of group‐level results using the nonparametric (permutation test) inference procedures available within the three software packages. For ds000120, a one‐sample repeated‐measure permutation test was not viable in AFNI, so nonparametric inference was excluded for this study.

The pipelines carried out for each study and software package are described in Section 2.2 of BMN, and a full decomposition of the modules used within each package is provided in Table 1 of the manuscript. Notably, we chose to implement a number of processing steps for all of our reanalyses regardless of whether they had been carried out in the original studies. These were procedures that we believed were fundamental to ensure our reproductions could be compared objectively, and steps that are widely considered as good practice within the community. Specifically, in all of our reanalyses we applied skull stripping to the T1‐weighted (T1w) structural image, we used each package's nonlinear registration tools to transform the structural and functional data to the anatomical template, and six motion regressors were included in the analysis design matrix for all pipelines (while more than six motion regressors are often used, we chose six as this could be easily implemented in all three software packages).

Here, our first aim was to isolate whether the largest variation between software occurs during the preprocessing or statistical modeling of the functional data. To add to the analyses that were conducted for BMN (where the entire workflow, including preprocessing, was carried out within each software package), we conducted a collection of similar pipelines except this time implementing *the same* preprocessing strategy to the three datasets before carrying out the rest of the analyses in the three packages. Comparisons of these sets of results would distinguish the impact each software package's preprocessing workflow can have on the final group‐level results.

For pipelines that used an identical preprocessing strategy, a common minimal preprocessing workflow was applied to each of the datasets using fMRIPrep 20.0.02 (Esteban et al., 2019, 2020; RRID:SCR_016216), which is based on Nipype 1.4.2 (Gorgolewski et al., 2011, Esteban et al., 2020; RRID:SCR_002502). The fMRIPrep pipeline combines procedures from a range of software packages to provide the optimal implementation at each stage of preprocessing. We will now describe the preprocessing sub‐workflows that were applied to all three datasets' anatomical and functional data within fMRIPrep. These pipelines are also summarized in Table 1, where we have included the tools implemented by fMRIPrep at each processing step. Notably, apart from a few procedures that relied on tools from FSL, most of the preprocessing performed by fMRIPrep used packages independent of AFNI, FSL, and SPM.

#### 2.2.1 Anatomical data preprocessing in fMRIPrep

For each of the three datasets, the preprocessing of the anatomical data was carried out within fMRIPrep as follows. The T1w image was corrected for intensity nonuniformity with N4BiasFieldCorrection (Tustison et al., 2010), distributed with ANTs 2.2.0 (Avants, Epstein, Grossman, & Gee, 2008, RRID:SCR_004757), and used as T1w‐reference throughout the workflow. The T1w‐reference was then skull‐stripped with a Nipype implementation of the antsBrainExtraction.sh workflow (from ANTs), using OASIS30ANTs as a target template. Brain tissue segmentation of cerebrospinal fluid (CSF), white‐matter (WM), and gray‐matter (GM) was performed on the brain‐extracted T1w using fast (FSL 5.0.9, RRID:SCR_002823, Zhang, Brady, & Smith, 2001). Brain surfaces were reconstructed using recon‐all (FreeSurfer 6.0.1, RRID:SCR_001847, Dale, Fischl, & Sereno, 1999), and the brain mask estimated previously was refined with a custom variation of the method to reconcile ANTs‐derived and FreeSurfer‐derived segmentations of the cortical GM of Mindboggle (RRID:SCR_002438, Klein et al., 2017). Volume‐based spatial normalization to one standard space (MNI152NLin2009cAsym) was performed through nonlinear registration with antsRegistration (ANTs 2.2.0), using brain‐extracted versions of both T1w reference and the T1w template. The following template was selected for spatial normalization: ICBM 152 Nonlinear Asymmetrical template version 2009c (Fonov, Evans, McKinstry, Almli, & Collins, 2009, RRID:SCR_008796; TemplateFlow ID: MNI152NLin2009cAsym).

#### 2.2.2 Functional data preprocessing in fMRIPrep

For each of the three datasets, the preprocessing of the functional data was carried out within fMRIPrep as follows. For each of the BOLD runs found per subject (across all tasks and sessions), the following preprocessing was performed. First, a reference volume and its skull‐stripped version were generated using a custom methodology of fMRIPrep. Susceptibility distortion correction (SDC) was omitted. The BOLD reference was then co‐registered to the T1w reference using bbregister (FreeSurfer) which implements boundary‐based registration (Greve & Fischl, 2009). Co‐registration was configured with six degrees of freedom. Head‐motion parameters with respect to the BOLD reference (transformation matrices, and six corresponding rotation and translation parameters) are estimated before any spatiotemporal filtering using mcflirt (FSL 5.0.9, Jenkinson, Bannister, Brady, & Smith, 2002). The BOLD time‐series (including slice‐timing correction when applied) were resampled onto their original, native space by applying the transforms to correct for head‐motion. These resampled BOLD time‐series will be referred to as preprocessed BOLD in original space, or just preprocessed BOLD. The BOLD time‐series were resampled into standard space, generating a preprocessed BOLD run in MNI152NLin2009cAsym space. First, a reference volume and its skull‐stripped version were generated using a custom methodology of fMRIPrep. Several confounding time‐series were calculated based on the preprocessed BOLD: framewise displacement (FD), DVARS and three region‐wise global signals. FD and DVARS are calculated for each functional run, both using their implementations in Nipype (following the definitions by Power et al., 2014). The three global signals are extracted within the CSF, the WM, and the whole‐brain masks. Additionally, a set of physiological regressors were extracted to allow for component‐based noise correction (CompCor, Behzadi, Restom, Liau, & Liu, 2007). Principal components are estimated after high‐pass filtering the preprocessed BOLD time‐series (using a discrete cosine filter with 128 s cut‐off) for the two CompCor variants: temporal (tCompCor) and anatomical (aCompCor). tCompCor components are then calculated from the top 5% variable voxels within a mask covering the subcortical regions. This subcortical mask is obtained by heavily eroding the brain mask, which ensures it does not include cortical GM regions. For aCompCor, components are calculated within the intersection of the aforementioned mask and the union of CSF and WM masks calculated in T1w space, after their projection to the native space of each functional run (using the inverse BOLD‐to‐T1w transformation). Components are also calculated separately within the WM and CSF masks. For each CompCor decomposition, the k components with the largest singular values are retained, such that the retained components' time series are sufficient to explain 50% of variance across the nuisance mask (CSF, WM, combined, or temporal). The remaining components are dropped from consideration. The head‐motion estimates calculated in the correction step were also placed within the corresponding confounds file. The confound time series derived from head motion estimates and global signals were expanded with the inclusion of temporal derivatives and quadratic terms for (Satterthwaite et al., 2013). Frames that exceeded a threshold of 0.5 mm FD or 1.5 standardized DVARS were annotated as motion outliers. All resamplings can be performed with a single interpolation step by composing all the pertinent transformations (i.e., head‐motion transform matrices, SDC when available, and co‐registrations to anatomical and output spaces). Gridded (volumetric) resamplings were performed using antsApplyTransforms (ANTs), configured with Lanczos interpolation to minimize the smoothing effects of other kernels (Lanczos, 1964). Nongridded (surface) resamplings were performed using mri_vol2surf (FreeSurfer).

### 2.3 Manipulation of modeling methods and hybrid pipeline generation

Alongside preprocessing, different parts of the three software packages' pipelines were interchanged to generate a collection of hybrid analysis pipelines. For these pipelines, AFNI (version AFNI_20.0.20), FSL (version 6.0.3), and SPM (standalone version of SPM12, r7771) were used. At the subject‐level, modeling was partitioned into three separate components: the fMRI signal model, noise model, and low‐frequency drift model. Specifically, the fMRI signal model concerns how each software package models the hemodynamic response function to obtain the task‐related regressors in the GLM, as well as how any parametric modulations or temporal derivatives are included in the GLM to model aspects of the BOLD response to the task conditions. The noise model pertains to how each package models temporal autocorrelation for prewhitening the fMRI data (Olszowy, Aston, Rua, & Williams, 2019), and the drift model concerns how each package implicitly includes predictors in the subject‐level GLM to regress out the low‐frequency drifts that are present in functional time‐series data (Smith et al., 1999). In addition to these subject‐level analysis components, we also interchanged between each package's group‐level and inference model.

Regarding implementation, for the subject‐level analyses it was not feasible to apply one software's noise model inside another package (e.g., it was not viable to conduct a workflow in SPM that implemented FSL's first‐level noise model). However, exchanging the fMRI signal and low‐frequency drift models between software could be done easily, by simply interchanging the relevant regressors in the design matrix. Because of this, for each of our hybrid analysis pipelines the choice of software used for modeling the noise ultimately determined the package through which the subject‐level analyses were conducted. For example, a hybrid pipeline using FSL's first‐level noise model with AFNI's first‐level fMRI signal model and drift model would be implemented within FSL, except the regressors in the design matrix for modeling the fMRI signal and low‐frequency drifts were then interchanged with the corresponding regressors from the design matrix generated by running the complete analysis within AFNI. In addition to this, six motion parameters (translations and rotations) estimated as part of the preprocessing workflow were included in all first‐level analysis models as nuisance regressors. Finally, for each hybrid pipeline the subject‐level contrast of parameter estimate maps were inputted into the software package specified by the workflow for group‐level analysis and inference.

**FIGURE 1:**
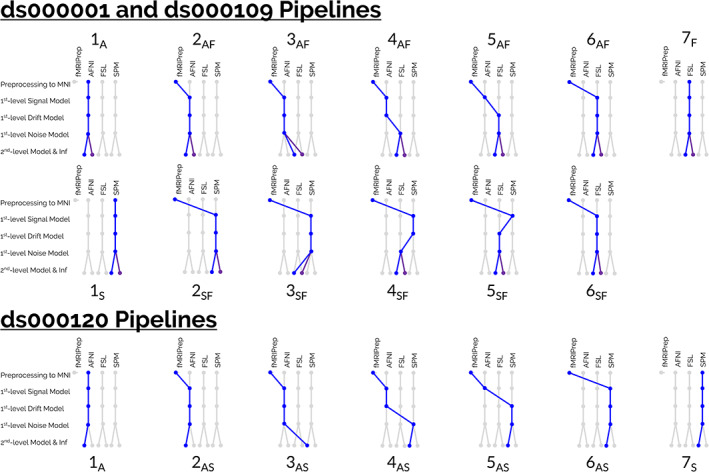
Diagrams to enumerate the complete set of 59 pipelines that were carried out on the three datasets. For ds000001 and ds000109, 26 pipelines were implemented for each dataset (13 workflows using parametric inference at the group‐level, and 13 parallel workflows using nonparametric inference displayed by the purple lines). For these two datasets, FSL was used as the reference software package, and hybrid pipelines were generated by interchanging procedures from either AFNI and FSL (**AF** pipelines) or SPM and FSL (**SF** pipelines) across the analysis workflow. A total of seven pipelines were carried out on the ds000120 dataset, where it was not feasible to analyze the data in FSL and nonparametric inference was unavailable in AFNI. Here, SPM was used as the reference software package, and hybrid pipelines were generated by interchanging procedures from AFNI and SPM across the analysis workflow. Notably, in this arrangement only one specific analysis procedure is changed between adjacent pipelines (from left‐to‐right). For instance, the only difference between the third and fourth pipelines on the top row was whether AFNI's or FSL's first‐level noise model was applied, and therefore any discrepancies between the group‐level results for these pipelines are wholly attributable to differences between the two softwares' noise models

Taking all combinations of software procedures considered across the three datasets yielded a total of 59 unique workflows, shown diagrammatically in Figure 1. The diagrams labeled **1** (far‐left) and **7** (far‐right) for each study display the pipelines that were carried out in BMN, where a single software package was used to conduct the entire analysis workflow (from preprocessing up to group analyses). Pipelines labeled **2** and **6** are similar, except that preprocessing was carried out using the fMRIPrep workflow described in the previous section. Pipelines labeled **3**–**5** include further manipulations, each step interchanging one aspect of the subject‐ and group‐level modeling as described above. For ds000001 and ds000109, modifications to the workflow were considered relative to the pipeline where the entire analysis was carried out within FSL (labeled **7F** in Figure 1, the “reference” pipeline). In other words, pipelines were generated by sequentially exchanging procedures from FSL with the corresponding procedures from AFNI (**AF** pipelines) or SPM (**SF** pipelines), as well as interchanging the preprocessing subflow with fMRIPrep. For ds000120, where group‐level analysis in FSL was not feasible, the pipeline carried out entirely in SPM (labeled **7S** in Figure 1) was used as the reference instead, and pipelines were generated by exchanging procedures from SPM with the corresponding procedures from AFNI. As previously discussed, for ds000001 and ds000109 we considered both parametric and nonparametric inference (purple lines in Figure 1) at the group‐level, while for ds000120 the repeated‐measures permutation test was not feasible in AFNI and therefore only parametric inference was considered.

Processing within AFNI was carried out using the “afni_proc.py” program. For ds000001 and ds000109, AFNI's “3dMEMA” program (Chen, Saad, Nath, Beauchamp, & Cox, 2012) was used for the parametric group‐level analyses, and “3dttest++” was used for the nonparametric group‐level analyses. For ds000120, “3dMVM” (Chen, Adleman, Saad, Leibenluft, & Cox, 2014) was used for the parametric group‐level analysis. Across all the studies, the “3dClustsim” method (Cox, Reynolds, & Taylor, 2016; Cox, Chen, Glen, Reynolds, & Taylor, 2017) was used for clustering the group‐level statistic maps. Subject‐ and group‐level analyses within FSL were carried out using FSL's FEAT (Woolrich, Behrens, Beckmann, Jenkinson, & Smith, 2004; Woolrich, Ripley, Brady, & Smith, 2001), and processing in SPM was implemented by specifying the relevant modules from the Batch Editor.

### 2.4 Comparison methods

Two comparison methods were considered to assess the nature of pipeline‐variability across each studies' collection of group‐level statistical results. Correlations (Pearson's *r*) were obtained for each pair of unthresholded group‐level statistic maps to evaluate differences in the overall activation profiles produced from each analysis workflow. As well as this, Dice coefficients were obtained for all pairwise combinations of thresholded statistic maps in order to compare the final locations of activation given by each analysis pipeline after correction for multiple comparisons. For a pair of thresholded maps, the Dice coefficient is calculated as the volume of the intersection of the two maps divided by the average of the volume's of each separate thresholded image. In other words, Dice measures the overlap of voxels between two sets of thresholded maps relative to the total spatial extent covered by both maps' activations (a Dice coefficient of 1 indicates identical locations of activation in both maps, while 0 indicates complete disagreement). With each Dice coefficient, the percentage of “spill‐over” activation was also computed, that is, the percentage of activation in one pipeline's thresholded statistic map that fell outside of the analysis mask of the other pipeline.

Finally, we applied a recently proposed image‐based meta‐analysis method that aggregated information across all pipelines (for a given dataset) to yield a “consensus” activation map, that is, the set of brain regions where significant activation was unanimous across *all* analysis pipelines applied to the data. The consensus analysis was performed on the collection of unthresholded group‐level *z*‐statistic images obtained across all pipelines, accounting for the correlations between pipelines owing to the same underlying data and identical procedures applied across parts of the analysis workflow. The method was originally proposed in Botvinik‐Nezer et al. (2020), where it was used to infer a consensus across results obtained by many analysis teams for a single task‐fMRI dataset. Full details of the method are provided in Section S8.1. We applied the consensus analysis methods to further examine the robustness of the individual results obtained for each dataset after accounting for the interpipeline variation.

For new analyses (all pipelines in Figure 1 excluding diagrams labeled **1** and **7** that were carried out as part of BMN), AFNI and FSL scripts were written in Python 3.7.6 and SPM scripts were written in GNU Octave (version 4.4.1). Scripts were made generalizable using a series of templates to extract the stimulus timings from the raw data, carry out the fMRIPrep preprocessing workflow, and subsequently conduct subject‐ and group‐level analyses. A master script for each dataset took the templates as inputs, replacing various holding variables to create distinct batch scripts for each of the unique pipelines. These batch scripts were subsequently executed within the master script to obtain all sets of group‐level results.

## 3 RESULTS

All analysis scripts and results have been made available, see the data availability statement for more details.

The preprocessing of each subject's data for all three studies was assessed using the summary reports provided as part of the fMRIPrep workflow. This included checking that each participant's functional data had been correctly masked and successfully registered to the MNI template image. Inspection of these reports confirmed that preprocessing had been successful for all‐but‐one subject, subject 4 from the ds000001 dataset. Exceptionally high intensities found in this subject's raw T1w anatomical image (potentially due to an erroneous brain‐extraction applied to the anatomical data before it was shared) caused drop‐out in sizable regions of the brain during bias‐field correction in the fMRIPrep preprocessing pipeline. This subsequently led to a highly shrunken brain mask, and failure to register this subject's functional data to the template image. For these reasons subject 4 was excluded from all further analyses, and we repeated all ds000001 analyses that were previously carried out for BMN with this subject removed. As such, all ds000001 results presented here are for 15 subjects rather than the complete sample of 16 whose data were originally shared.

### 3.1 Main sources of pipeline‐variability

A detailed review of the regions of activation found in the thresholded maps across all pipelines for each dataset is provided in Section S8.2. Further Supporting Information figures (including slice views of the thresholded and unthresholded results maps obtained from all pipelines) are provided in Section S8.3. Here we describe the main sources of variation observed between the pipelines' results across the three datasets.

**FIGURE 2:**
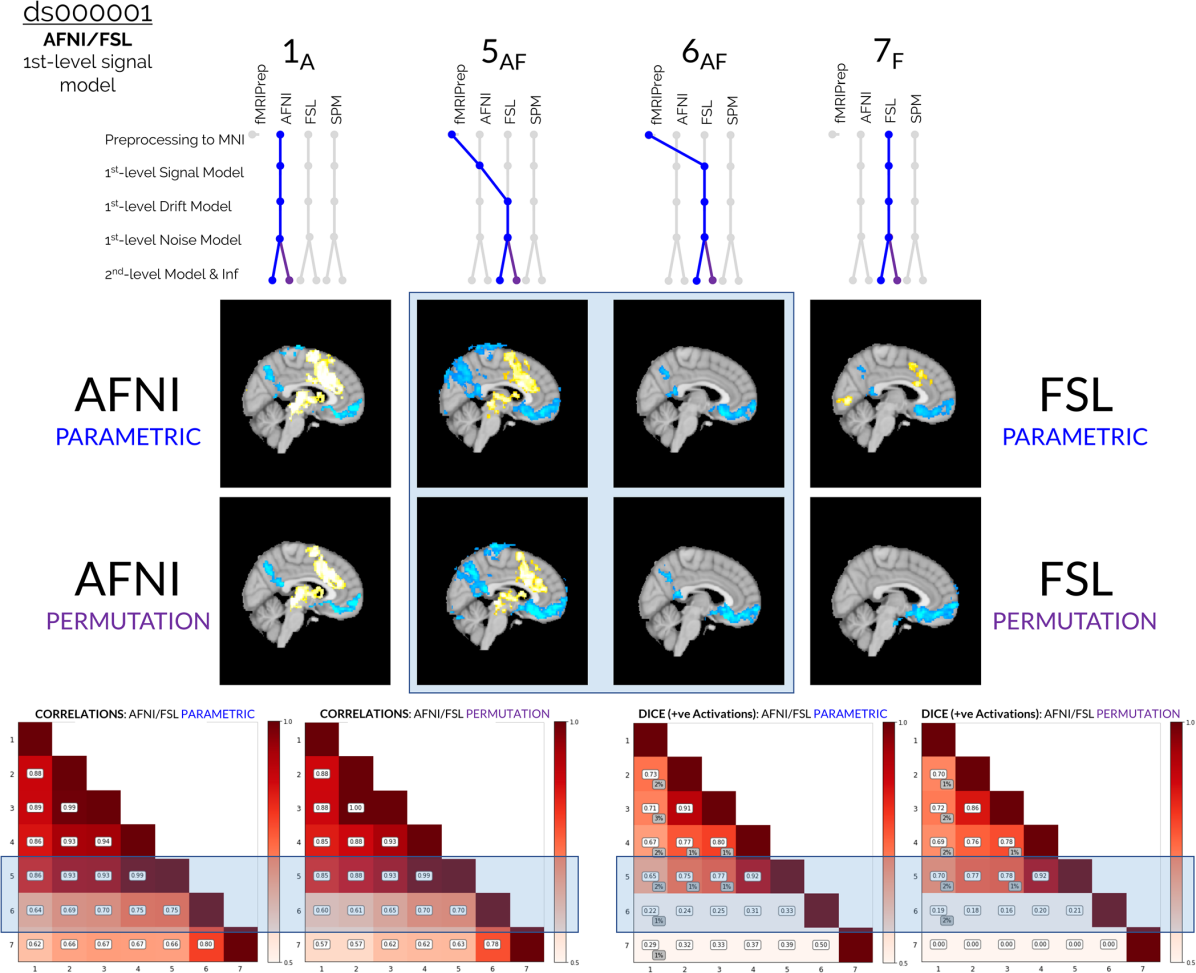
Comparisons of the group‐level thresholded *t*‐statistic maps (cluster‐forming threshold *p* <.01, clusterwise threshold *p <*.05 FWE‐corrected), correlation values, and Dice coefficients obtained from reanalyses of the ds000001 dataset, focusing on the collection of results obtained from hybrid pipelines that implemented procedures from both AFNI and FSL. The axis labels on the correlation and Dice plots here correspond with the pipeline labels given in Figure 1, that is, the label “1” on the correlation and Dice plot axes here correspond to the results obtained from pipeline **1A** shown in Figure 1. Blue windows highlight the disagreement between the two sets of results given by pipelines **5AF** and **6AF**, which differed only as to whether AFNI's or FSL's first‐level signal model was used. The interchange of the signal model between packages led to more expansive differences in the final results than any other individual processing step: in the thresholded group‐level *t*‐statistic maps (middle), the expansive clusters of positive activation in the anterior cingulate (among other brain regions) identified by the pipeline using AFNI's signal model (workflow **5AF**) were lost when interchanged with FSL's signal model (workflow **6AF**). Differences in the thresholded maps are also reflected in the Dice coefficient matrices (bottom right), where the Dice values dramatically fell due the change of signal model between pipelines **5AF** and **6AF**. The moderate decreases also seen in the correlation values for these two pipelines (bottom‐left) indicate that the interchange of signal model led to a considerable difference in the overall activation profile of the unthresholded *t*‐statistic image

Our analyses of the ds000001 dataset suggest that differences in each software's first‐level signal model were the largest contributor to variation across the three software packages' final statistical results. This is highlighted in Figures 2 and S1 (all Supporting Information figures described in this section can be found in Section S8.3), where in both figures we compare the results from the two analysis workflows which differed only by the choice of first‐level signal model: In Figure 2, pipeline **5SF** applied SPM's first‐level signal model while pipeline **6SF** applied FSL's; in Figure S1, pipeline **5AF** applied AFNI's signal model while pipeline **6AF** applied FSL's. In both cases, switching to FSL's signal model led to a considerable change in the final thresholded results, evidenced by the sizable difference in the Dice coefficients obtained for these two specific workflows (highlighted by the blue windows in the Dice plots for Figures 2 and S1, bottom right). Particularly, a large cluster of positive activation observed in the anterior cingulate for the pipelines implementing SPM's and AFNI's first‐level signal model was not identified in the corresponding set of thresholded results that used FSL's signal model (Figures 2 and S1, thresholded maps, middle). However, these changes were not simply caused by subtle differences magnified by the thresholding, as considerable decreases in the correlation values for the unthresholded maps can also be observed for these two workflows (Figures 2 and S1, correlation plots, bottom left), indicating that dissimilarities between the signal model's applied by the two pipelines ultimately led to radically different activation profiles in the unthresholded group‐level *t*‐statistic images.

In terms of preprocessing, the results from our analyses across all studies indicate that AFNI's preprocessing pipeline was the most similar of the three software packages to fMRIPrep, while FSL's was the least similar. Evidence for this is seen in Figures S2 and S3, where we compare results obtained for ds000001 and ds000109, respectively, for two pairs of workflows: pipelines **1A** and **2AF**, which differ only as to whether fMRIPrep or AFNI's preprocessing procedure was carried out, and pipelines **6AF** and **7F**, which differ only as to whether fMRIPrep or FSL's preprocessing was applied. In each plot, differences between the results from pipelines **1A** and **2AF** have been highlighted with a blue window, while differences between **6AF** and **7F** have been highlighted with a green window. In all cases, it can be seen that the final results obtained with either fMRIPrep or AFNI's preprocessing workflow had greater comparability than the corresponding fMRIPrep/FSL results: the final thresholded activation clusters for fMRIPrep/AFNI pipelines were more similar relative to the fMRIPrep/FSL thresholded results (Figures S2 and S3, middle plots), and the correlation and Dice coefficients comparing pipelines **1A** and **2AF** were consistently larger than the corresponding values for pipelines **6AF** and **7F** (Figures S2 and S3, bottom‐left and bottom‐right plots). The fMRIPrep/SPM Dice and correlation values can be seen in Figures S9, S10, S13, S14, and S15; on the whole, these are slightly better than the corresponding FSL values, and slightly worse than the AFNI figures.

**FIGURE 3:**
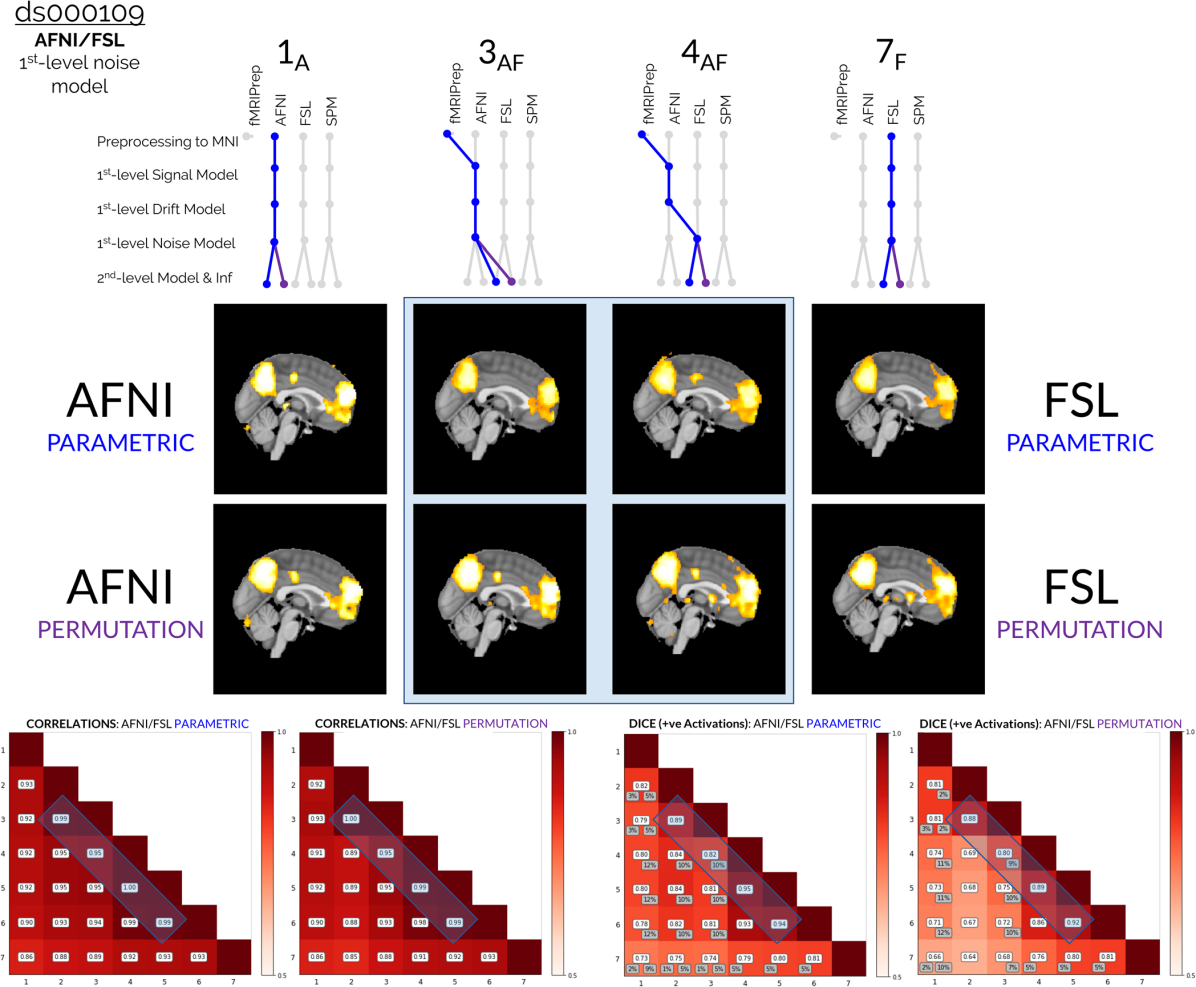
Comparisons of the group‐level thresholded *t*‐statistic maps (cluster‐forming threshold *p* <.005, clusterwise threshold *p <*.05 FWE‐corrected), correlation values, and Dice coefficients obtained from reanalyses of the ds000109 dataset, focusing on the collection of results obtained from hybrid pipelines that implemented procedures from both AFNI and FSL. The two sets of results given by pipelines **3AF** and **4AF** are displayed, which differed only as to whether AFNI's or FSL's first‐level noise model was implemented. Preprocessing aside, the interchange of the first‐level noise model impacted the final group‐level results more than any other modeling decision. This is highlighted in the correlation and Dice plots at the bottom of the figure: the blue windows on the off‐diagonals show that the pairwise correlation and Dice values for pipelines **3AF** and **4AF** are smaller than the corresponding values obtained for all other pairs of adjacent pipelines. In the thresholded *t*‐statistic maps (blue window, middle), it can be seen that while both of these workflows captured the main effects in the precuneus and frontal brain areas, pipeline **4AF** (that used FSL's first‐level noise model) also determined numerous smaller activation clusters which were not captured by pipeline **3AF** (that used AFNI's noise model)

Aside from preprocessing, the single analysis step that caused the most variation in the ds000109 results was the first‐level noise model. In Figures 3 and S4, we focus our attention on how changing from AFNI's first‐level noise model (Figure 3) or SPM's first‐level noise model (Figure S4) to FSL's noise model caused a more considerable change in the final results relative to the other processing steps. In both figures, it can be seen that the correlation values (Figures 3 and S4, bottom left plots) and Dice values (Figures 3 and S4, bottom right plots) obtained for comparisons between pipelines **3** and **4** (which differ only by choice of first‐level noise model) were generally worse than all other comparisons of pipelines varying by a single analysis step (values magnified by the blue windows in the bottom plots). However, it is notable that all correlations and Dice values were greater than 0.8 here, and the overall variation between results for ds000109 was much less than that observed for ds000001.

**FIGURE 4:**
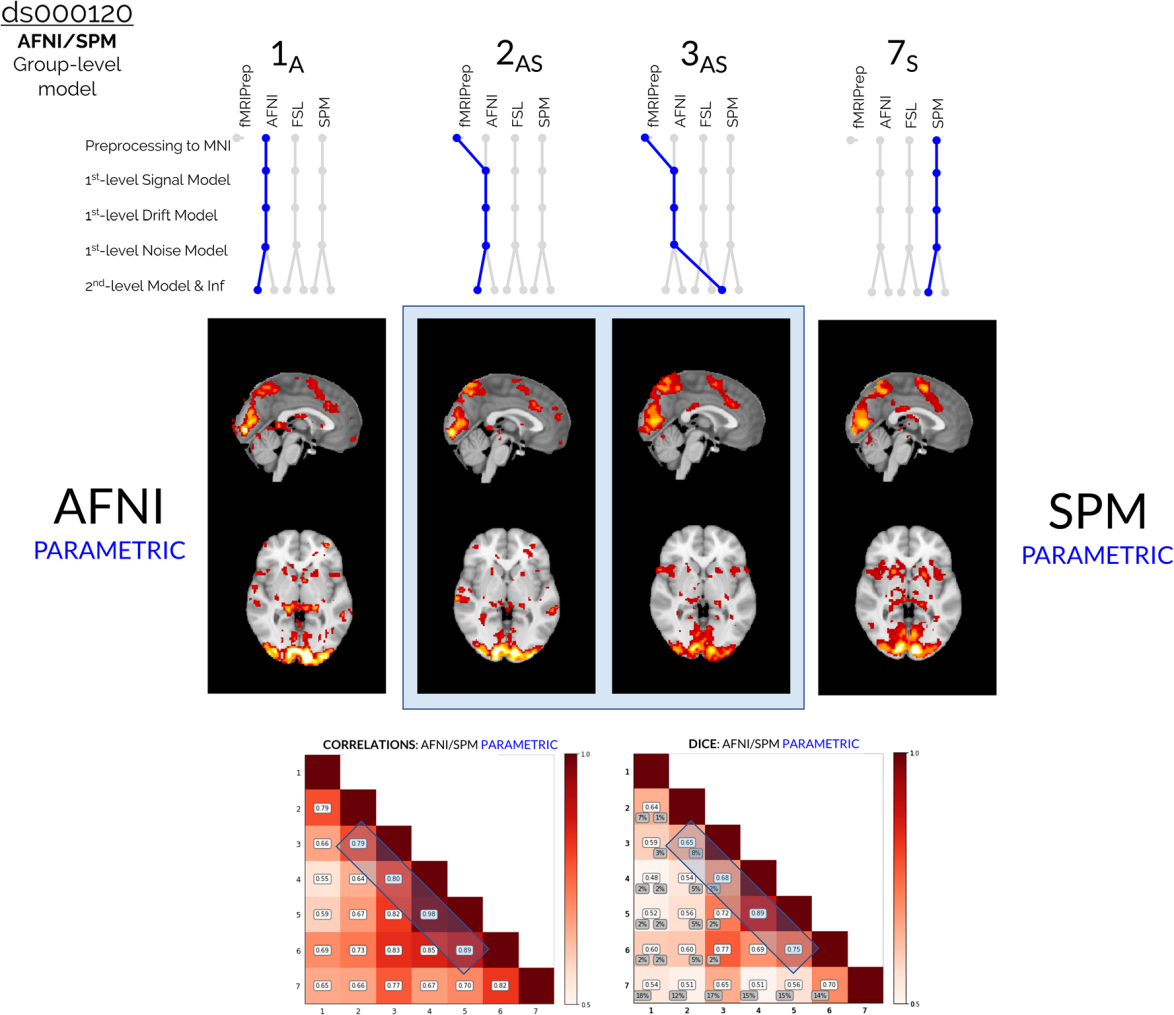
Comparisons of the group‐level thresholded *F*‐statistic maps (cluster‐forming threshold *p <*.001, clusterwise threshold *p* <.05 FWE‐corrected), correlation values, and Dice coefficients obtained from reanalyses of the ds000120 dataset, focusing on the collection of results obtained from hybrid pipelines that implemented procedures from both AFNI and SPM. The two sets of results given by pipelines **2AS** and **3AS** are displayed, which differed only as to whether AFNI's or SPM's group‐level inference model was implemented. The interchange of the second‐level model impacted the final results more than any other modeling decision. This is highlighted in the correlation and Dice plots at the bottom of the figure: the blue windows on the off‐diagonals show that the pairwise correlation and Dice values for pipelines **2AS** and **3AS** are smaller than the corresponding values obtained for all other pairs of adjacent pipelines. In the thresholded *F*‐statistic maps (blue window, middle), while both pipelines captured the main effects in the visual cortex, pipeline **2AS** (that used AFNI's group‐level model and inference) identified more smaller clusters scattered across central brain regions compared to pipeline **3AS** (that used SPM's group‐level model and inference). It can also be seen that AFNI's inference model reported larger *F*‐statistic values in activated regions compared to SPM

For ds000120, the group‐level model and inference procedure was the largest source of variability between software. This is seen in Figure 4, where the two analysis workflows which differed only by the choice of group‐level inference model are compared: pipeline **2AS** applying AFNI's group‐level modeling and inference, and pipeline **3AS** applying SPM's group‐level modeling and inference. Similar to ds000109, while the main effects were captured in the thresholded *F*‐statistic maps by both packages (for ds000120, both **2AS** and **3AS** identified large clusters in the visual cortex), there was more disagreement over the presence of weaker effects. In this case, pipeline **2AS** (that used AFNI's group‐level inference model) determined a greater quantity of smaller clusters scattered across central regions of the brain compared to pipeline **3AS** (that used SPM's group model). It is also notable that AFNI's group‐level model generally determined larger *F*‐statistic values in the main activated regions compared to SPM (higher statistic values in the visual cortex for pipelines **1A** and **2AS** compared to **3AS** and **7S** in Figure 4).

Finally, we observed that the choice as to which software's first‐level drift model was applied in the analysis pipeline led to minimal changes in the final analysis results. This is shown in Figures S5 and S6, where we highlight the similarity in results obtained for ds000001 and ds000109, respectively, for two workflows (pipelines **4SF** and **5SF**) which only differed as to whether SPM or FSL was used to model the drift. In both figures, it can be seen that the thresholded results obtained for these two pipelines (Figures S5 and S6, middle plots) were qualitatively very similar, that the unthresholded maps obtained with these two workflows were almost perfectly correlated (Figures S5 and S6, bottom‐left plots), and that Dice comparisons for the thresholded maps were consistently around 90%.

### 3.2 Consensus analyses

**FIGURE 5:**
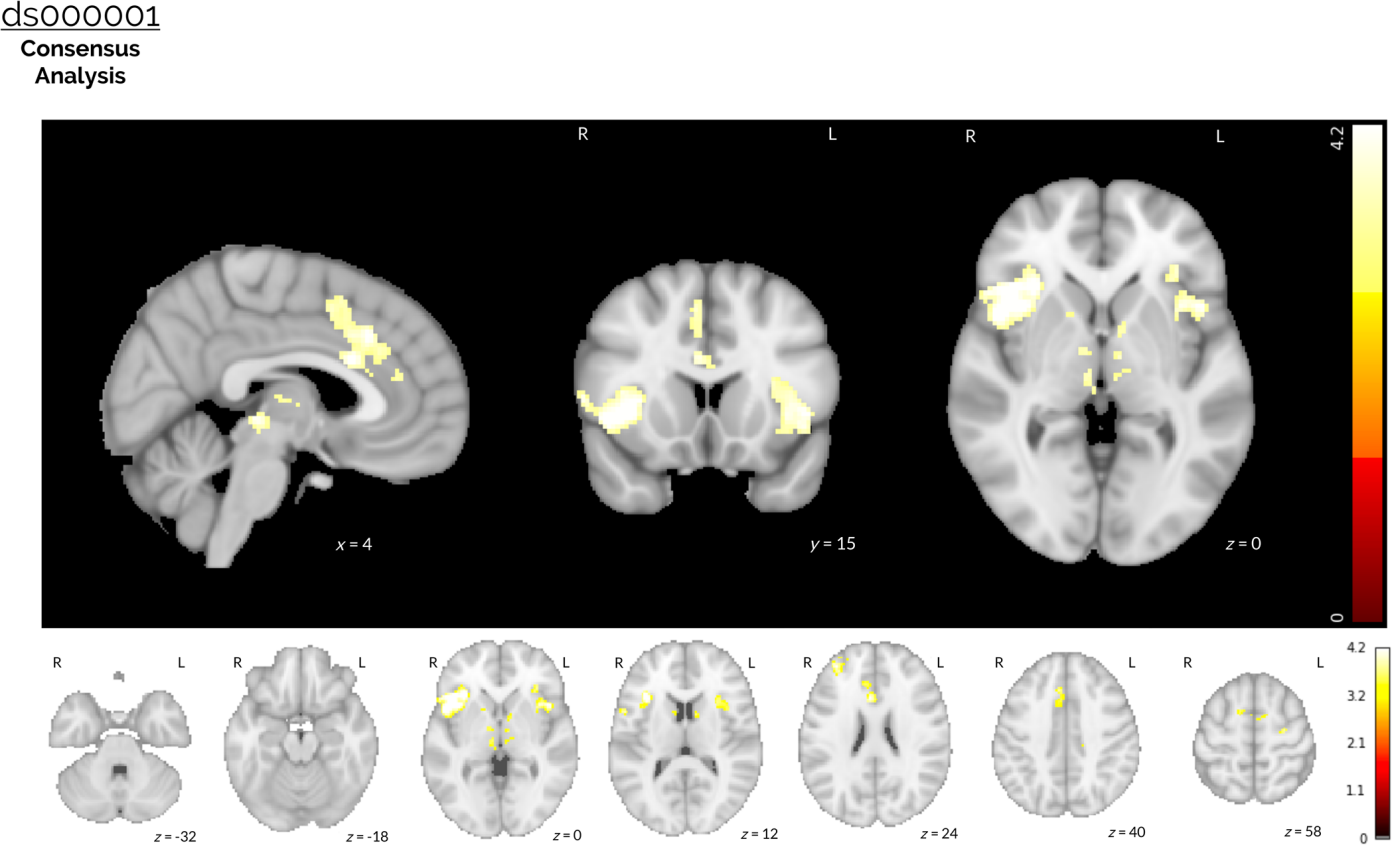
Results of the ds000001 image‐based meta‐analysis. A consensus analysis was performed on the unthresholded *z*‐statistic maps obtained from all 26 pipelines used to analyze the ds000001 dataset, accounting for the correlation between pipelines owing to the same underlying data and identical procedures implemented across parts of the analysis workflow. The thresholded *z*‐statistic map displayed shows voxels for which the group‐level consensus statistic was significantly greater than zero after a voxelwise FDR correction (*p <*.05). Activation was found in the anterior cingulate, the insular cortex (bilateral) and the inferior frontal gyrus (right side only) after accounting for between‐pipeline variation. No (negative) activation was obtained when the equivalent inference was performed to determine voxels where the consensus statistic was significantly *less* than zero

**FIGURE 6:**
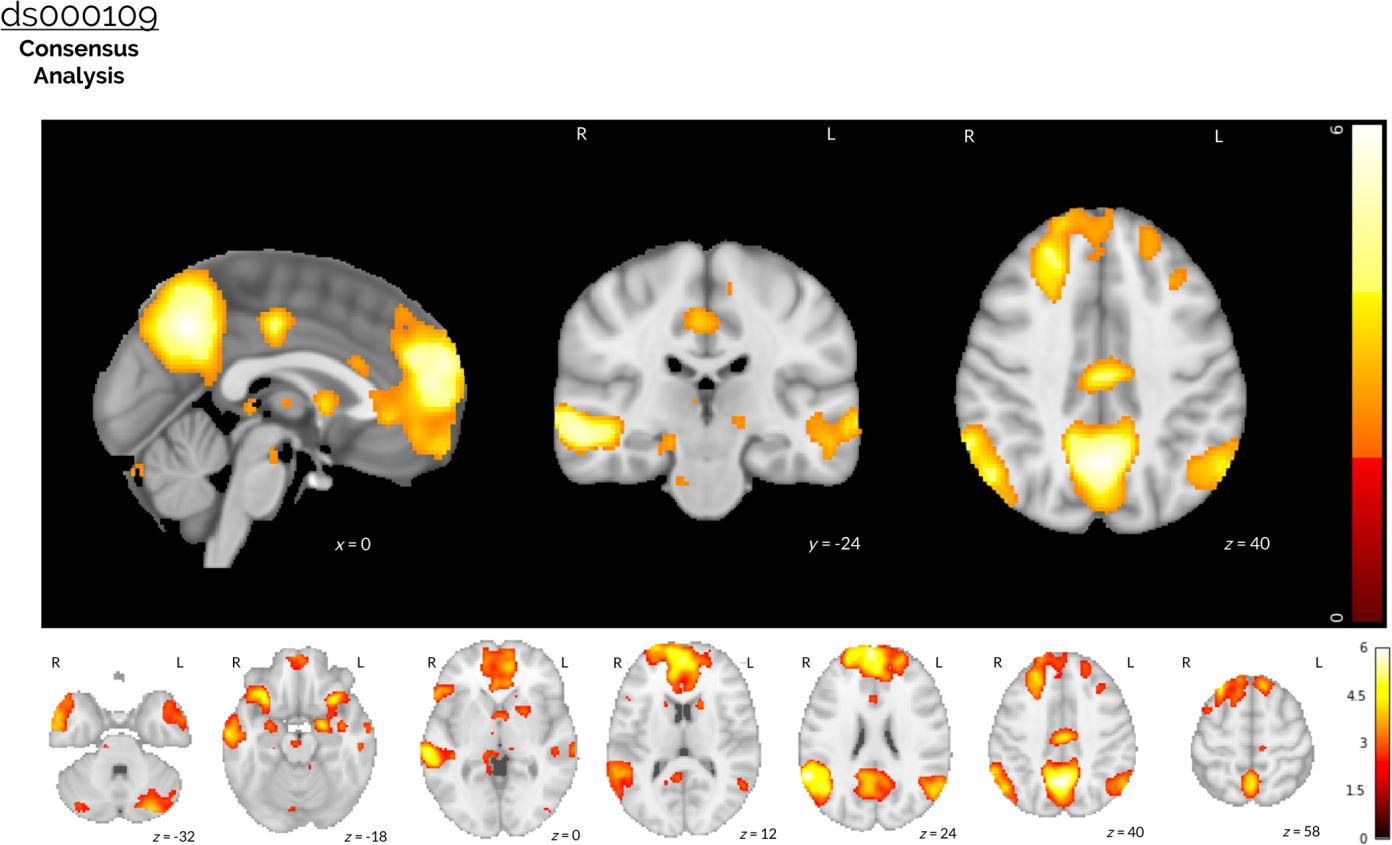
Results of the ds000109 image‐based meta‐analysis. A consensus analysis was performed on the unthresholded *z*‐statistic statistical maps obtained from all 26 pipelines used to analyze the ds000109 dataset, accounting for the correlation between pipelines owing to the same underlying data and identical procedures implemented across parts of the analysis workflow. The thresholded *z*‐statistic map displayed shows voxels for which the group‐level consensus statistic was significantly greater than zero after a voxelwise FDR correction (*p* <.05). Large activation clusters included areas of the precuneus, frontal pole and superior frontal gyrus, the bilateral superior occipital cortex and angular gyri (bilateral). Further activation was found in the middle temporal gyrus (posterior and anterior divisions, bilateral), the left and right amygdalae, and the posterior cingulate gyrus. No (negative) activation was obtained when the equivalent inference was performed to determine voxels where the consensus statistic was significantly *less* than zero

Slice views of the thresholded *z*‐statistic maps from the consensus analyses performed on the ds000001 and ds000109 datasets are presented in Figures 5 and 6, respectively. For each dataset, the consensus analysis took the form of an image‐based meta‐analysis conducted on the unthresholded group‐level *z*‐statistic maps obtained from *all* 26 pipelines through which the data had been analyzed. The image‐based meta‐analysis computed a third‐level *z*‐statistic map, where each statistic value in the image reflected the level of evidence to which all pipelines had agreed activation was present at a given voxel. This map was then thresholded to determine voxels for which the consensus *z*‐statistic was significantly greater than zero after a voxelwise FDR correction (*p <*.05). The equivalent one‐sided correction was also performed to determine voxels whose consensus statistic was significantly *less* than zero.

For ds000001, the thresholded *z*‐statistic image presented in Figure 5 shows a consensus across pipelines of positive activation in the anterior cingulate, the insular cortex (bilateral) and the inferior frontal gyrus (right side only). Significant activation was also determined in these brain areas for nearly all of the thresholded group‐level *t*‐statistic maps obtained from each individual analysis workflow, as can be seen in Figures S7–S10. However, the thresholded *z*‐statistic for a consensus on negative activations failed to determine any brain areas that were statistically significant after FDR correction.

For ds000109, the thresholded *z*‐statistic image presented in Figure 6 shows a consensus across pipelines of positive activation in a variety of brain regions. Large activation clusters covered areas of the precuneus, frontal pole and superior frontal gyrus, the bilateral superior occipital cortex and angular gyri (bilateral), and further activation was determined in the middle temporal gyrus (posterior and anterior divisions, bilateral), the left and right amygdalae, and the posterior cingulate gyrus. The main effects seen here were also captured in all of the thresholded group‐level *t*‐statistic maps obtained from each individual analysis workflow, displayed in Figures S11–S14. Once again, the thresholded *z*‐statistic for a consensus on negative activations failed to determine any brain areas that were statistically significant after the voxelwise correction.

## 4 DISCUSSION

Comparisons of the statistical maps obtained from the collection of pipelines applied to the three datasets have shown both the robustness and fragility of group‐level task‐fMRI results in response to variation of the software package at different stages of the analysis workflow. While results were found to be highly stable across all datasets when the analysis package used to model the low‐frequency fMRI drifts was interchanged, other analytic manipulations produced more appreciable changes in the group‐level results. For instance, the final regions of activation obtained for the ds000001 dataset were found to be highly contingent on the software package used to model the fMRI signal; switching between AFNI/FSL's signal model (pipelines **5AF** and **6AF** in Figure 2) and SPM/FSL's signal model (pipelines **5SF** and **6SF** in Figure S1) both produced considerable differences in the *t*‐statistic maps (Dice coefficients less than .35 for comparisons of the thresholded maps, correlations less than .75 for unthresholded maps). However, for ds000109 the change of signal model had minimal impact on the final results (in Figure S11, comparisons of the **5AF** and **6AF** unthresholded maps yielded a correlation of .99, Dice coefficient of .94 for positive activations). In contrast, the interchange of ds000109's first‐level noise model produced greater relative differences, and for ds000120, the group‐level model was found to be the largest modeling source of between‐software variability (Figures 3, S4, and S11–S15).

Importantly, these results do not provide an indication as to which software package is better or worse; without a gold standard to compare to, no such claims can be made. However, these findings do suggest that the main sources of software‐variability across the analysis pipeline can be heterogeneous and dependent on external factors such as the analysis design or task paradigm under investigation. One reason that the quantitative comparisons for the ds000001 dataset were generally worse than the corresponding ds000109 and ds000120 comparisons is likely to be due to the smaller sample size for this study (15 for ds000001 vs. 21 and 17 for ds000109 and ds000120, respectively). As well as this, the larger impact of the signal model for ds000001 may be attributed to varying aspects between the three studies' analysis designs. In particular, the event‐related design used for ds000001's balloon analog risk task could have been more sensitive to differences between each package's hemodynamic response model compared to the block design used for ds000109. In addition to this, while ds000109 and ds000120 did not apply any modulated regressor orthogonalization methods, for each of the three ds000001 task events represented in the GLM (pumps, cash‐outs, and explosions) the response time regressors were orthogonalized with respect to the average activity regressor (e.g., *pumps*_*response_time*_ condition orthogonalized with respect to *pumps*_*average*_ condition). It has been previously observed in the fMRI literature that the three software packages handle orthogonalization differently (Mumford, Poline, & Poldrack, 2015): while in FSL each regressor can be orthogonalized with respect to any other individual regressor the user has specified, in SPM orthogonalization is applied automatically, and each regressor is orthogonalized with respect to *all* other conditions preceding it in the model. We suspect that differences in the shared variance between regressors caused by divergent orthogonalization procedures across the three packages is one of the reasons that the choice of signal model proved to be so influential for ds0000001.

The inference procedure carried out specifically for ds000001 may have also contributed to variation in the activation clusters identified in the thresholded *t*‐statistic maps, particularly for this study's collection of parametric inference results. Group‐level inference was conducted using a cluster‐forming threshold of *p* <.01 uncorrected for the ds000001 study. However, in Eklund, Nichols, and Knutsson (2016) it was found that parametric clusterwise inference using this cluster‐forming threshold led to varying degrees of inflated false activations across the three software packages. Notably, while these findings may in‐part explain the poor Dice coefficients for the ds000001 parametric results, they do not have any bearing on the correlation comparisons (since correlations were comparisons of the *unthresholded* maps) or the collection of corresponding nonparametric results (since permutation inference was shown to perform as expected in Eklund et al.). These findings also do not affect the ds000001 consensus analysis results in Section 3.2, which used a voxelwise FDR correction for inference. Since the correlation and nonparametric inference comparisons were observed to be poorer for ds000001 compared to the other studies too, this could suggest that divergence between the parametric results were also caused by other factors than the cluster‐forming threshold.

Our findings for the ds000109 dataset, where the choice of first‐level noise model had the largest impact on the results relative to any other processing step, are supported by recent research where AFNI, FSL, and SPM's autocorrelation models were empirically assessed using resting‐state and task fMRI data. In Olszowy et al., 2019, it was found that the performance of the three package's noise models could vary depending on the dataset and task‐paradigm under investigation, and specifically, that low‐frequency block designs were affected the most. Since ds000109 was the only study we reanalyzed using a block design, this could explain why the choice of noise model was most influential here.

Our findings for the ds000120 dataset, where the group‐level model had the largest influence on the final statistical results, may be explained by differences in how AFNI and SPM model the repeated‐measures design that was used for this study. Specifically, while SPM's “full factorial design” module assumes that the variance–covariance structure of the repeated measures is uniform across all voxels, and estimates these correlations by pooling across “similar” voxels (i.e., activated voxels that are spatially close; Glaser & Friston, 2004), AFNI's “3dMVM” computes these correlations separately at each voxel instead (Chen et al., 2014). The divergence between these two approaches may partially explain the larger values seen in the thresholded *F*‐statistic maps for pipelines that used AFNI's group‐level inference model (compared to SPM) in Figures 4 and S15.

From the quantitative comparisons presented for all studies, it is notable how seemingly small differences in the unthresholded maps could be amplified after thresholding. Even when pairwise correlations of the unthresholded statistic maps were considerably high, in many cases the corresponding Dice comparisons measuring the overlap of activation in the thresholded maps were substantially lower. This is illustrated by the comparisons of pipelines **6AF** and **7F** in Figures S3 and S11, where the correlation between these two pipelines' unthresholded *t*‐statistic maps was .93, but the Dice coefficient for negative activations in the thresholded maps was 0. Ultimately, this was due to pipeline **6AF** identifying two clusters of negative activation in the left and right interior temporal gyri, while pipeline **7F** did not determine any negative activation. The overriding issue here is the dichotomous nature of thresholding; because maps are binarized into regions of activation and nonactivation based on a single cut‐off value, substantially different thresholded maps can be obtained depending on whether a cluster's size is marginally above or below the threshold. We believe this scenario demonstrates why the unthresholded statistical maps should always be shared. Access to the unthresholded maps enables further meta‐analyses of the data to be conducted, where the variation of clusters across diverse samples (and analysis workflows) can be quantified in order to determine where results converge. The consensus analyses carried out as part of this work exemplify the benefits to such an approach, and notably the thresholded consensus map for ds000109 (Figure 6) did not identify any regions of negative activation after accounting for the interpipeline variation between individual results.

One limitation of this work pertains to the sample sizes for the three studies that have been reanalyzed. In total, for the ds000001, ds000109, and ds000120 datasets respectively, our reanalyses used data from 15, 21, and 17 participants. While these sample sizes may be small, they are fairly representative of the typical samples that have been used in task‐fMRI studies up to this point (fig. 1 of Poldrack et al., 2017 estimates that the median sample size for task‐fMRI studies was below 20 until the last decade). Nonetheless, a further assessment of the effects of analytic variability in response to increasing samples sizes would be a valuable addition to the literature. The growing availability of “big” task‐fMRI datasets is providing greater opportunities to carry out such an assessment.

Another limitation is the small number of studies that have been reanalyzed in this work. Due to the restrictive requirements of our study (and particularly, the need for task‐based fMRI data with analysis methods compatible within AFNI, FSL, and SPM), the three studies examined here were the only datasets hosted on OpenNeuro deemed suitable for extensive multi‐software analyses at the onset of our investigation. Nevertheless, a larger sample of studies will need to be analyzed to provide a further understanding of how variation in fMRI results caused by differences in analysis software generalizes across diverse datasets and task designs. Alongside this, there are various analysis parameters that were not explored in this work, including different registration methods (all our reanalyses applied nonlinear registration to the MNI template), different voxel sizes, further thresholding methods (e.g., FDR, FSL's threshold‐free cluster enhancement (Smith & Nichols, 2009) and AFNI's equitable thresholding and clustering (Cox, 2019), and two‐tailed testing (Chen et al., 2019). We are optimistic that increased data‐sharing efforts and further development of libraries that provide unified interfaces to different software packages (e.g., NiPype, Gorgolewski et al., 2011) will help to facilitate a more comprehensive exploration of analytic variability in the field going forward.

In conclusion, we believe that multi‐software analyses are essential to understanding the nature and origins of intersoftware differences. For pipeline elements that produce the greatest variation, further study will be required to determine an optimal or preferred method (Churchill, Spring, Afshin‐Pour, Dong, & Strother, 2015). However, until more research on pipeline harmonization has been carried out it is important that individual task‐fMRI datasets are analyzed using a range of plausible workflows (and software packages), and possibly by many analysis teams. To obtain multiple results from one dataset, individual research teams may pursue a “multiverse” analysis strategy (Simonsohn, Simmons, & Nelson, 2019; Steegen, Tuerlinckx, Gelman, & Vanpaemel, 2016), where the raw data are analyzed using a number of feasible workflows (as has been done in this work). By deriving numerous analysis results from a single dataset, meta‐analytic methods can then be applied to account for the variability between pipelines and integrate inconsistent findings. In this regard, we hope that the consensus analysis approach utilized in this study (that also considers the dependencies between different pipelines) can provide researchers with a viable option here. Alongside the multiverse approach, numerous results can also be obtained from one dataset through traditional replication analyses. To this end, it is vital that both practitioners and publishers embrace the importance of replication studies and the publication of null findings. Alongside this, replication can only become possible if data sharing practices become commonplace in the field. In this work, we have shared all of our statistical results (both unthresholded and thresholded maps) and analysis code via public online repositories (Neurovault and Github/Zenodo), and we hope that other researchers will follow suit to advance transparency in neuroimaging science.

## Supporting information


**Figure S1**: Similar to Figure 2, except this time focusing on the collection of results obtained from hybrid pipelines that implemented procedures from both SPM and FSL (rather than AFNI and FSL). Once again, the interchange of first‐level signal model led to more extensive differences in the final results than any other individual processing stop, and similar to Figure 2, this was largely due to the complete loss of positive activation in the thresholded maps that occurred when SPM's first‐level signal model (pipeline **5SF**) was interchanged with FSL's first‐level signal model (pipeline **6SF**). Relative to the corresponding AFNI/FSL correlations presented in Figure 2, the correlation values connected to pipeline **6SF** are improved here (bottom‐left). This suggests that the overall differences in the activation profiles of the unthresholded maps for pipelines **5SF** and **6SF** were more subtle compared to the corresponding AFNI/FSL results, but that these differences were amplified after the FWE clusterwise correction was applied to obtain the thresholded maps.
**Figure S2**: Comparisons of the group‐level thresholded *t*‐statistic maps (cluster‐forming threshold *p* <.01, clusterwise threshold *p* <.05 FWE‐corrected), correlation values, and Dice coefficients obtained from reanalyses of the ds000001 dataset. Blue windows compare the two sets of results obtained from pipelines **1A** and **2AF**, which differed only as to whether preprocessing was carried out within AFNI or within fMRIPrep, respectively. Green windows compare pipelines **6AF** and **7F**, which differed only as to whether preprocessing was carried out within FSL or fMRIPrep. Qualitative and quantitative comparisons displayed here show a high degree of similarity between the two sets of results where either AFNI or fMRIPrep preprocessing was used, while greater differences can be seen for the two pipelines where FSL's preprocessing workflow was interchanged with fMRIPrep. In particular, the slice views of the thresholded *t*‐statistic maps for pipelines **1A** and **2AF** look strikingly similar (middle, blue window), while disagreement can be seen in terms of the brain regions that were positively activated for the parametric results obtained with pipelines **6AF** and **7F** (middle, green window). Alongside this, the correlation and Dice values for the pairwise comparisons of the **1A** and **2AF** results (blue windows, bottom‐left and bottom‐right) were better than the corresponding values obtained for **6AF** and **7F** regardless of whether parametric or nonparametric inference was performed.
**Figure S3**: Similar to Figure S2, except this time focusing on the corresponding pipeline results for reanalyses of the ds000109 dataset. While greater similarity can be seen on‐the‐whole for the ds000109 results compared to ds000001, it is notable that there was still disagreement between pipelines **6AF** and **7F** in terms of the negatively activated brain regions for the parametric inference results: while the thresholded results for pipeline **6AF** (that used fMRIPrep's preprocessing workflow) determined two clusters of negative activation in the inferior temporal gyrus (bilateral), pipeline **7F** (identical to **6AF** except that FSL's preprocessing was used) did not determine any negative activation. Consequently, the Dice coefficient for comparisons of these two pipelines is zero (green window in the blue negative activation Dice matrix at bottom).
**Figure S4**: Similar to Figure 3, except this time focusing on the collection of results obtained from hybrid pipelines that implemented procedures from both SPM and FSL (rather than AFNI and FSL). Once again, setting preprocessing aside, the interchange of the first‐level noise model between pipelines **3SF** and **4SF** led to more extensive differences in the results than any other modeling procedure. This is highlighted in the blue windows on the off‐diagonals of the correlation and Dice matrices at the bottom of the figure, where the values for pipelines **3SF** and **4SF** can be seen to be lower than the corresponding values for other pairs of adjacent pipelines in most cases. Similarly to Figure 3, the thresholded *t*‐statistic map for pipeline **4SF** (that used FSL's first‐level noise model) determined slightly more smaller activation clusters than the corresponding set of results for pipeline **3SF** (that used SPM's noise model).
**Figure S5**: Comparisons of the group‐level thresholded *t*‐statistic maps (cluster‐forming threshold *p* <.01, clusterwise threshold *p* <.05 FWE‐corrected), correlation values, and Dice coefficients obtained from reanalyses of the ds000001 dataset, focusing on the collection of results obtained from hybrid pipelines that implemented procedures from both SPM and FSL. The two sets of results given by pipelines **4SF** and **5SF** are displayed, which differed only as to whether SPM's or FSL's first‐level drift model was used. Overall, the change of drift model had minimal impact on the final results; pairwise comparisons show that the unthresholded maps for pipelines **4SF** and **5SF** were almost perfectly correlated for both the parametric and nonparametric inference cases (bottom‐left, blue windows). The Dice values are marginally worse (bottom‐right, blue window)—close to 90%—due to slightly more negative activation determined by pipeline **5SF** (that used FSL's first‐level drift model) as seen in the thresholded *t*‐statistic maps (middle, blue window). Nevertheless, the correlations and Dice comparisons for pipelines **4SF** and **5SF** are the best of all pairs of adjacent pipelines.
**Figure S6**: Similar to Figure S5, except this time focusing on the corresponding pipeline results for reanalyses of the ds000109 dataset. Once again, the correlations (blue windows, bottomleft) and Dice values (blue windows, bottom‐right) for pairwise comparisons of pipelines **4SF** and **5SF** were some of the best of any, indicating that the interchange of drift model between SPM and FSL minimally impacted the final group‐level results. In the thresholded *t*‐statistic images (blue window, middle), it can be seen that the change of drift model between the two software packages only led to slight changes in the locations of some of the smaller activation clusters.
**Figure S7**: ds000001 AFNI/FSL pipelines (parametric results). Comparisons of the group‐level thresholded *t*‐statistic maps (cluster‐forming threshold *p* <.01, clusterwise threshold *p* <.05 FWE‐corrected), correlation values, and Dice coefficients obtained from reanalyses of the ds000001 dataset. The collection of all parametric inference results obtained from hybrid pipelines that implemented procedures from both AFNI and FSL are presented.
**Figure S8**: ds000001 SPM/FSL pipelines (parametric results). Comparisons of the group‐level thresholded *t*‐statistic maps (cluster‐forming threshold *p* <.01, clusterwise threshold *p* <.05 FWE‐corrected), correlation values, and Dice coefficients obtained from reanalyses of the ds000001 dataset. The collection of all results obtained from hybrid pipelines that implemented procedures from both AFNI and FSL are presented.
**Figure S9**: ds000001 AFNI/FSL pipelines (nonparametric results). Comparisons of the group‐level thresholded *t*‐statistic maps (cluster‐forming threshold *p* <.01, clusterwise threshold *p* <.05 FWE‐corrected), correlation values, and Dice coefficients obtained from reanalyses of the ds000001 dataset. The collection of all nonparametric inference (permutation test) results obtained from hybrid pipelines that implemented procedures from both AFNI and FSL are presented.
**Figure S10**: ds000001 SPM/FSL pipelines (nonparametric results). Comparisons of the group‐level thresholded *t*‐statistic maps (cluster‐forming threshold *p* <.01, clusterwise threshold *p* <.05 FWE‐corrected), correlation values, and Dice coefficients obtained from reanalyses of the ds000001 dataset. The collection of all nonparametric inference (permutation test) results obtained from hybrid pipelines that implemented procedures from both SPM and FSL are presented.
**Figure S11**: ds000109 AFNI/FSL pipelines (parametric results). Comparisons of the group‐level thresholded *t*‐statistic maps (cluster‐forming threshold *p* <.005, clusterwise threshold *p* <.05 FWE‐corrected), correlation values, and Dice coefficients obtained from reanalfrom both AFNI and FSL are presented. Yses of the ds000109 dataset. The collection of all parametric inference results obtained from hybrid pipelines that implemented procedures from both AFNI and FSL are presented.
**Figure S12**: ds000109 AFNI/FSL pipelines (nonparametric results). Comparisons of the group‐level thresholded *t*‐statistic maps (cluster‐forming threshold *p* <.005, clusterwise threshold *p* <.05 FWE‐corrected), correlation values, and Dice coefficients obtained from reanalyses of the ds000109 dataset. The collection of all nonparametric inference (permutation test) results obtained from hybrid pipelines that implemented procedures from both AFNI and FSL are presented.
**Figure S13**: ds000109 AFNI/FSL pipelines (nonparametric results). Comparisons of the group‐level thresholded *t*‐statistic maps (cluster‐forming threshold *p* <.005, clusterwise threshold *p* <.05 FWE‐corrected), correlation values, and Dice coefficients obtained from reimplemented procedures from both AFNI and FSL are presented. Analyses of the ds000109 dataset. The collection of all nonparametric inference (permutation test) results obtained from hybrid pipelines that implemented procedures from both AFNI and FSL are presented.
**Figure S14**: ds000109 SPM/FSL pipelines (nonparametric results). Comparisons of the group‐level thresholded *t*‐statistic maps (cluster‐forming threshold *p* <.005, clusterwise threshold *p* <.05 FWE‐corrected), correlation values, and Dice coefficients obtained from reanalyses of the ds000109 dataset. The collection of all nonparametric inference (permutation test) results obtained from hybrid pipelines that implemented procedures from both SPM and FSL are presented.
**Figure S15**: ds000120 AFNI/SPM pipelines (parametric results). Comparisons of the group‐level thresholded *F*‐statistic maps (cluster‐forming threshold *p* <.001, clusterwise threshold *p* <.05 FWE‐corrected), correlation values, and Dice coefficients obtained from reanalfrom both AFNI and SPM are presented. Yses of the ds000120 dataset. The collection of all parametric inference results obtained from hybrid pipelines that implemented procedures
**Figure S16**: ds000001 AFNI/FSL pipelines (parametric results). Comparisons of the group‐level unthresholded *t*‐statistic maps obtained from reanalyses of the ds000001 dataset. The collection of all parametric inference results obtained from hybrid pipelines that implemented procedures from both AFNI and FSL are presented.
**Figure S17**: ds000001 SPM/FSL pipelines (parametric results). Comparisons of the group‐level unthresholded *t*‐statistic maps obtained from reanalyses of the ds000001 dataset. The collection of all parametric inference results obtained from hybrid pipelines that implemented procedures from both SPM and FSL are presented.
**Figure S18**: ds000001 AFNI/FSL pipelines (nonparametric results). Comparisons of the group‐level unthresholded *t*‐statistic maps obtained from reanalyses of the ds000001 dataset. The collection of all nonparametric inference (permutation test) results obtained from hybrid pipelines that implemented procedures from both AFNI and FSL are presented.
**Figure S19**: ds000001 SPM/FSL pipelines (nonparametric results). Comparisons of the group‐level unthresholded *t*‐statistic maps obtained from reanalyses of the ds000001 dataset. The collection of all nonparametric inference (permutation test) results obtained from hybrid pipelines that implemented procedures from both SPM and FSL are presented.
**Figure S20**: ds000109 AFNI/FSL pipelines (parametric results). Comparisons of the group‐level unthresholded *t*‐statistic maps obtained from reanalyses of the ds000109 dataset. The collection of all parametric inference results obtained from hybrid pipelines that implemented procedures from both AFNI and FSL are presented.
**Figure S21**: ds000109 SPM/FSL pipelines (parametric results). Comparisons of the group‐level unthresholded *t*‐statistic maps obtained from reanalyses of the ds000109 dataset. The collection of all parametric inference results obtained from hybrid pipelines that implemented procedures from both SPM and FSL are presented.
**Figure S22**: ds000109 AFNI/FSL Pipelines (nonparametric results). Comparisons of the group‐level unthresholded *t*‐statistic maps obtained from reanalyses of the ds000109 dataset. The collection of all nonparametric inference (permutation test) results obtained from hybrid pipelines that implemented procedures from both AFNI and FSL are presented.
**Figure S23**: ds000109 SPM/FSL pipelines (nonparametric results). Comparisons of the group‐level unthresholded *t*‐statistic maps obtained from reanalyses of the ds000109 dataset. The collection of all nonparametric inference (permutation test) results obtained from hybrid pipelines that implemented procedures from both SPM and FSL are presented.
**Figure S24**: ds000120 AFNI/SPM pipelines (nonparametric results). Comparisons of the group‐level unthresholded F‐statistic maps obtained from reanalyses of the ds000120 dataset. The collection of all parametric inference results obtained from hybrid pipelines that implemented procedures from both AFNI and SPM are presented.

## Data Availability

All scripts and results have been made available through our Open Science Framework (OSF; Foster & Deardorff, 2017) Project at https://osf.io/axy3w/ (Bowring, Nichols, & Camille, 2021), and all group‐level statistic maps have been made available on NeuroVault: https://neurovault.org/collections/8381/, https://neurovault.org/collections/7113/, https://neurovault.org/collections/9324/, for ds0000001, ds000109, and ds000120, respectively. Python Jupyter Notebooks have also been shared for each of the three studies, harvesting the results data from Neurovault and applying the comparison methods discussed in the previous section to create all the figures used here. All analysis scripts, results reports, and notebooks for each study are available through Zenodo (Nielsen, Smith, Erdmann, & Simko, 2014) at http://doi.org/10.5281/zenodo.5070414.
